# Developing and Characterization of a Biopolymeric Membrane Derived from Mature Banana Peel Biomass

**DOI:** 10.3390/polym17060775

**Published:** 2025-03-14

**Authors:** Aramis A. Sánchez, Ana Guamán, Darwin Castillo, Javier Carrión, Grettel Riofrío, J. P. Padilla-Martínez, Vasudevan Lakshminarayanan

**Affiliations:** 1 PROSUR Construcción Sustentable del sur, Puebla 72810, Mexico; aramis.scientist@gmail.com; 2 Department of Chemistry, Universidad Técnica Particular de Loja, Loja 1101608, Ecuador; jfcarrion1@utpl.edu.ec (J.C.); gariofrio1@utpl.edu.ec (G.R.); 3 Faculty of Animal and Biological Sciences, Universidad Técnica Estatal de Quevedo, Quevedo 120550, Ecuador; aguamang4@uteq.edu.ec; 4 Theoretical and Experimental Epistemology Lab, School of Optometry and Vision Science, University of Waterloo, 200 University Avenue West, Waterloo, ON N2L 3G1, Canada; vengulak@uwaterloo.ca; 5 Institute of Sciences, Benemérita Universidad Autónoma de Puebla, Puebla 72960, Mexico; juan.padilla@correo.buap.mx; 6 Departments of Physics, Electrical and Computer Engineering and Systems Design Engineering, University of Waterloo, 200 University Avenue West, Waterloo, ON N2L 3G1, Canada

**Keywords:** biodegradable, mechanical resistance, absorbance, banana, biomass, biopolymeric

## Abstract

Biopolymeric films derived from starch are gaining attention due to their potential applications, which are primarily attributed to their availability and biodegradability. Here, we report developing and characterizing a biopolymeric film utilizing banana peel waste (BM2). Analytical techniques were employed, including water absorption analysis, determination of soluble matter, UV-visible absorption spectrophotometry, tensile strength assessment, morphological examination using scanning electron microscopy (SEM), and thermal analysis through thermogravimetric analysis (TGA). The water absorption analysis revealed a noteworthy absorption percentage of 115.23% and 61.75% of soluble matter. The UV-visible absorption spectrophotometry results demonstrated a light absorbance degree ranging from 0.9 to 720 nm, particularly between 400 and 1000 nm. However, the mechanical strength tests indicated relatively low resistance at 0.8 MPa, attributed to the irregular surface observed in the film’s morphology as evidenced by scanning electron microscopy (SEM). Thermal analysis conducted via TGA offered valuable insights into the degradation behavior of the film. The findings reveal a degradation temperature ranging from 160 to 300 °C, thereby elucidating the thermal stability of the film and its potential applications. While mechanical limitations were evident, the biopolymeric film derived from banana peel waste demonstrated noteworthy water absorption properties, presenting potential in specific applications, particularly those that do not necessitate elevated mechanical strength. Continued efforts in optimizing and refining the film’s structure promise to bolster its mechanical properties, making it suitable for various applications.

## 1. Introduction

Nowadays, the increasing accumulation of plastic waste, predominantly derived from non-biodegradable petroleum-based polymers, poses a critical environmental challenge. Approximately 150 million tons of plastic are produced annually, and those conventional plastics persist in the environment for extended periods, contributing to soil and water contamination, disrupting ecosystems, and potentially impacting human health [1].

Despite mitigation strategies such as recycling and incineration, these methods often fail to provide a long-term solution and may perpetuate environmental damage. In this context, biodegradable polymers [2,3] derived from renewable resources constitute one alternative to the solution to the mentioned problem; for example, natural biopolymers such as starch, cellulose, and proteins have gained attention due to their biodegradability, biocompatibility, and widespread availability [4,5,6,7,8].

Due to their ability to form film-like structures upon gelatinization, low cost, and abundance in nature, starch-based bioplastics are particularly attractive to research and the industry [8,9,10]. Several studies have explored using starch in creating membranes due to its widespread availability, cost-effectiveness, and gelatinization capability when exposed to high temperatures and water [11]. Starch comprises glucose units known as amylose and amylopectin in varying amounts depending on the source. These molecules are responsible for the gelatinization process of starch. However, films derived from starch often exhibit high water sensitivity and limited mechanical strength compared to synthetic plastics [12].

Therefore, one favorable alternative to research and the production of biodegradable films is the use of agricultural waste, such as fruit peels [12]. Several types of fruits, including mangoes, oranges, and bananas, contain significant amounts of starch compounds and biological activity that can be used to produce biodegradable materials [13,14]. Among these materials, banana peels are notable for their high carbohydrate and phytochemical content, which includes polyphenols, anthocyanins, tocopherols, phytosterols, and ascorbic acid, which contribute to improving the enhance the film’s functionality and antioxidant properties. 

The composition of mature banana peel (*Musa paradisiaca* and *Musa cavendish* species) differs depending on factors such as cultivar, ripeness stage, and environmental conditions [15,16]. The structural backbone of banana peel is composed of polysaccharides, starch, cellulose, and pectin, making it a practical raw material for creating biodegradable films [17]. 

Proximate analysis of mature banana peel reveals that it is composed of starch (~3.5–6.3%), resistant starch (~2.3–2.5%), dietary fiber (~47–53%), carbohydrates (~60–76%), crude fat (~2–11%), protein (~5.5–9%), lipids (~1–3%), and ash (~9–11%), along with significant amounts of potassium (K), calcium (Ca), and magnesium (Mg) [16,18]. Moreover, the banana peel contains antioxidants such as polyphenols, flavonoids, and carotenoids [19], which can improve the functional properties of biopolymeric membranes [7,20,21], principally in food packaging and biomedical applications [16,22,23].

Using mature banana peels as a biopolymeric feedstock presents multiple advantages [6,20,21,24,25,26]: (i) It offers a low-cost and sustainable alternative by repurposing agricultural waste [27,28]. (ii) The high carbohydrate content allows for effective starch extraction, making it a viable precursor for film formation. (iii) Including natural antioxidants can enhance film stability and extend food shelf life in packaging applications [20].

Several studies [3,22,23,29,30] have explored the potential of banana peel-derived biopolymers, yet research focusing on the systematic characterization of these films’ mechanical, thermal, and morphological properties remains limited. Furthermore, while banana peel-based films have been proposed for packaging, a detailed evaluation of their feasibility in industrial applications is still needed [9,31].

Despite these advantages, banana peel-based films have limitations, including high water absorption, moderate mechanical strength, and irregular morphology due to undissolved cellulose fibers [12,17]. To improve film performance, addressing these challenges requires optimizing formulation parameters, such as plasticizer content and processing conditions [23,28]. Concerning biodegradability, such films biodegrade faster than traditional plastics; however, they may degrade too quickly under high-moisture conditions [15].

While banana peel-based bioplastics have been explored in several works [15,17,22,23,28,32], there is a gap in the research regarding their performance in membrane form for packaging applications. In this context, this project presents the development and characterization process of a biopolymeric membrane (BM2) derived from mature banana peel biomass, aiming to enhance the performance of starch-based biodegradable films.

Therefore, this study aims to (i) develop and characterize a biopolymeric membrane derived from mature banana peel biomass and (ii) evaluate its mechanical properties, thermal stability (TGA analysis), and morphological properties (SEM analysis) to assess its suitability for sustainable applications. The results from this project are intended to contribute to developing an environmentally friendly and biodegradable alternative to conventional food packaging materials and bridge the gap between experimental biopolymer formulation and the real world.

This paper is structured as follows: The methodology section overviews the process of obtaining the banana film and the different steps to measure its properties. The results and discussion section shows the evaluation of the experimental film characteristics, comparisons, and values. Finally, a conclusion section of this work is presented.

## 2. Materials and Methods

### 2.1. Film Formation

**Figure 1:**
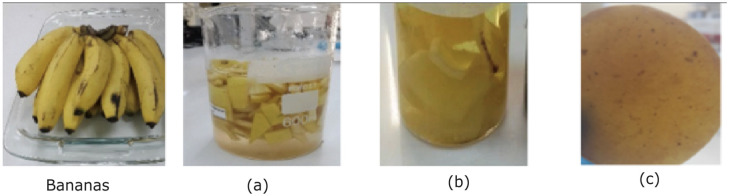
Process of Banana Film (*M. acuminata* L.): (**a**) mature banana peel fragments were immersed in a 50 mL sodium metabisulfite solution; (**b**) cooking process at 110 °C for 25 min under continuous magnetic stirring; and (**c**) biopolymeric membrane derived from banana.

To obtain the film (called BM2), 50 g of small banana peel fragments were submerged in 1% sodium metabisulfite to prevent the natural oxidation of the sample (see Figure 1a). The mixture and solid were then boiled with 70 mL of distilled water at 110 °C for 10 min with occasional stirring. Subsequently, the mixture was blended for 5 min to achieve a homogeneous paste (see Figure 1b).

The resulting paste was then placed into a second stage, where it was cooked at 110 °C for 25 min under continuous magnetic stirring. According to the methodology proposed by [12], this stage involved the addition of 2 mL each of 0.5 N HCl 37% (Thermo Fisher Scientific- Fisher Chemical brand, Geel, Belgium), 0.5 N NaOH (Scharlab, Sentmenat, Spain), and 2 mL of a plasticizer (glycerin 99.88%) (Merck KGaA, Darmstadt, Germany) per 50 g of sample. These additions aimed to optimize the biopolymeric material’s characteristics [21]. 

Finally, the mixture was carefully poured and spread into Petri dishes and subjected to a drying process in an oven set at 45 °C for 48 h. The dried films were kept for further analysis.

This careful procedure ensures the production of a high-quality biopolymeric membrane derived from the mature banana peel, ready for subsequent characterization studies (see Figure 1c).

### 2.2. Film Solubility in Water and Moisture Uptake

The biopolymeric films’ water absorption capacity and moisture uptake were evaluated following the ASTM [33] standard method. 

On the other hand, solubility was assessed based on the decrease in the sample’s weight in relation to the immersion time in the water to which it was subjected.

To initiate the experiment, three biopolymer sheets were conditioned in an oven set at 50 °C for 24 h. Following this conditioning period, the sheets were promptly transferred to a desiccator for 15 min to achieve equilibrium and were then accurately weighed (the recorded weight corresponds to the conditioned weight). Subsequently, these conditioned samples were immersed in beakers containing 100 mL of distilled water for two hours, ensuring complete submersion. After immersion, the water was removed using absorbent paper, and the samples were reweighed (the recorded weight corresponded to the wet weight). With these data, the water absorption percentage of the biopolymer was calculated.

The wet samples from the previous process were submerged in 100 mL of distilled water for an additional 24 h to determine the amount of soluble matter. They were then reconditioned, drying the samples in an oven at 45 °C for 24 h.

The membranes underwent a reconditioning phase, allowing them to dry for 24 h at a controlled temperature of 45 °C. Finally, the samples were cooled within a desiccator and subjected to a final weighing.

This systematic methodology provides a comprehensive understanding of the film’s behavior in water, allowing for precise quantification of water absorption and soluble matter content. 

### 2.3. UV-Visible Spectrophotometry

The assessment of film transparency was conducted through UV-visible spectrophotometry, a crucial step in understanding the optical characteristics of the biopolymeric films, as outlined by [7]. This technique provides valuable insights into how the films interact with light across a broad spectrum.

The UV-visible spectra were measured using a Thorlabs CCS200 (Newton, NJ, USA) spectrophotometer, covering the wavelength range from 200 to 1000 nm. This wide range allows for a detailed examination of the film’s transparency and its behavior across various wavelengths of light. The obtained spectra offer information on factors such as light absorbance and transmittance, contributing to a comprehensive understanding of the optical properties of the biopolymeric film.

### 2.4. Mechanical Properties

The evaluation of the mechanical properties of biopolymeric films is critical to gauge their structural integrity and suitability for various applications. The measurement of mechanical resistance was conducted following the protocol suggested by [14] and adhering to the ASTM [34] standard method.

(1) $$E=\frac{\sigma }{\varepsilon }=\frac{F/A}{dl/l}$$

Equation (1) describes Young’s modulus (E) as a property of the material that tells us how easily it can stretch and deform. It is defined as the ratio of tensile stress (σ) to tensile strain (ε), where stress is the amount of force applied per unit area (σ=F/A) and strain is extension per unit length (ε=dl/l).

To measure tensile strength (TS), elongation, and Young’s modulus (see Equation (1)), a Brookfield CT3 texture analyzer (Texture Analyzer, AMETEK Brookfield, Middleboro, MA, USA) was used. The samples were cut into strips of 80 mm in length and 25 mm in width according to the ASTM D88 [14] protocol and were conditioned at 25 °C in a desiccator for 7 days to prevent measurement errors due to ambient moisture absorption [8]. The strip ends were clamped for the test, ensuring an initial separation of 50 mm. Finally, the force and elongation until rupture were recorded.

### 2.5. Scanning Electron Microscopy (SEM)

The film’s surface morphology was visualized using scanning electron microscopy (SEM), utilizing the advanced Bruker VEGA 3 TESCAN instrument (Scanning Electron Microscope (SEM), TESCAN, Brno, Czech Republic) with an energy-dispersive X-ray spectrometer (EDS). The SEM analysis was conducted at an acceleration voltage of 20 kV at the Universidad Nacional del Chimborazo (UNACH).

The samples were cut into square segments measuring approximately 0.25 cm and carefully mounted on a sample holder using double-sided carbon tape. A thin layer of gold coating was applied using an SPI Module Sputter Coater from the United States for 40 s to enhance conductivity and visualization.

### 2.6. Thermogravimetric Analysis (TGA)

The thermal stability evaluation was determined using a differential scanning calorimeter DSC-TGA SDT Q600 V8.3 Build 101(Simultaneous Thermogravimetric Analyzer and Differential Scanning Calorimeter (TGA/DSC), TA Instruments, New Castle, DE, USA), employing the ramp method under a nitrogen atmosphere (N_2_). Film samples weighing 15.496 mg were scanned from room temperature (30 °C) to 1000 °C at a heating rate of 20 °C/min. 

The use of an inert atmosphere, specifically nitrogen (N_2_), is paramount in preventing oxidation or combustion during the analysis, considering the organic nature of the material. To ensure the optimal conditions for the experiment, a constant nitrogen flow rate of 100 mL/min was maintained throughout the analysis.

## 3. Results and Discussions

### 3.1. Water Absorption and Solubility

The biopolymeric film obtained from the banana peel (BM2) exhibited notable water absorption characteristics, registering a percentage of 115.23% with a corresponding soluble matter content of 61.75%. This water absorption level surpassed soy films, which typically fall within the range of 63–75% water absorption [13]. Understanding and comparing water absorption properties was pivotal in determining the film’s suitability for various applications.

The interaction between plasticizer, biopolymer chains, and water is enhanced under specific drying conditions, rendering the films hydrophilic [35]. The soluble nature of BM2 dried at 45 °C with 50% relative humidity can be attributed to such favorable conditions.

According to [36], plasticizers increase solubility by reducing interactions between biopolymeric molecules, making them more hydrophilic. The low molecular weight of the plasticizer facilitates its insertion between biopolymer chains, further enhancing solubility [36]. The solubility characteristics observed in BM2 underscore the importance of plasticizer selection and processing conditions in tailoring the film’s properties.

Additionally, the affinity of biopolymer membranes, particularly those based on starches, for water can accelerate the degradation of certain hydrophobic biopolymers [37]. Consequently, in some cases, blending starches with non-degradable polymers is recommended to balance the solubility and degradation characteristics. As exhibited by BM2, high solubility could make it well-suited for applications such as packaging wraps, where controlled solubility can offer specific advantages. The nuanced understanding of water absorption and solubility presented here is crucial for determining the potential applications and optimizing the performance of the biopolymeric film derived from banana peel.

Comparing these results with other biopolymeric films (see Table 1), the BM2 (our banana peel-based biopolymeric film) exhibited higher water absorption and solubility than other films based on banana and corn starch [17,19,28,32]. The BM2 membrane demonstrates high water absorption, a common characteristic of starch-based biopolymers. However, further modifications such as crosslinking, hydrophobic coatings, or nanofiller incorporation may be necessary to enhance its moisture resistance for practical packaging applications.

### 3.2. UV-Visible Spectrophotometry Results

**Figure 2:**
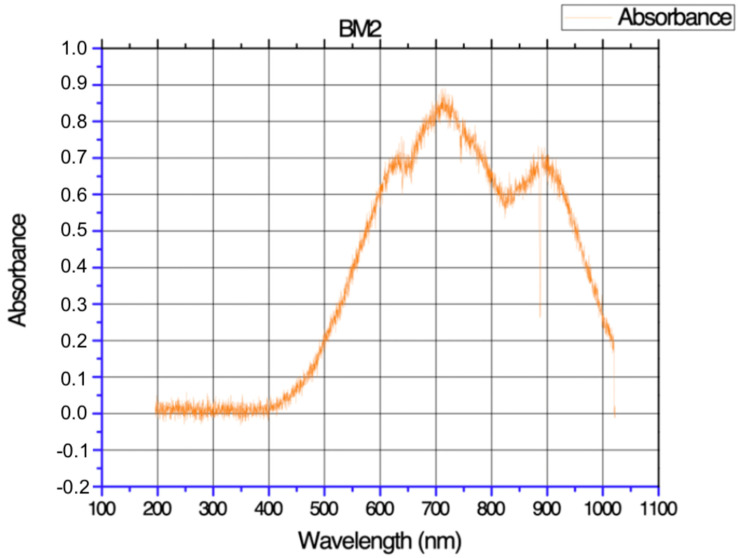
UV-Vis absorbance spectrum of the Banana peel film. The biopolymeric membrane (BM2) shows a maximal absorbance at 720 nm with an absorbance value of 0.9, indicating the film’s key molecular interactions and optical properties.

The absorption characteristics of the biopolymeric film are shown in Figure 2. The white light used for the analysis covered a wavelength range from 200 to 1000 nm.

The observed spectrum reveals insightful details about the film’s optical properties. Notably, the maximum wavelength (*λ_max_*) absorbed by the film molecules is recorded at 720 nm, with an absorbance value of 0.9. This absorption peak falls within the infrared spectral region. The first peak, with an absorbance of 0.75, is situated within the visible spectrum in the range of 625 nm. The second peak, exhibiting an absorbance of 0.72, occurs near the infrared region, specifically around 890 nm [38]. This dual-peak pattern suggests the film’s responsiveness to visible and infrared light.

Understanding the absorbance peaks is vital for potential applications, as it provides insights into the film’s transparency and how it interacts with different regions of the electromagnetic spectrum. 

### 3.3. Mechanical Properties Results

To examine the mechanical properties of biopolymer membranes derived from banana peel, the tensile strength of BM2 was analyzed, showing the load-bearing capacity of the biopolymeric membrane as it responded to an applied load.

The maximum load capacity was 2.347 kg. From these values, it was determined that the maximum tensile stress sustained by the material before failure was 0.028 MPa (28 kPa). The tensile strength value may be attributed to the high starch content present in banana peel [20]. 

**Figure 3:**
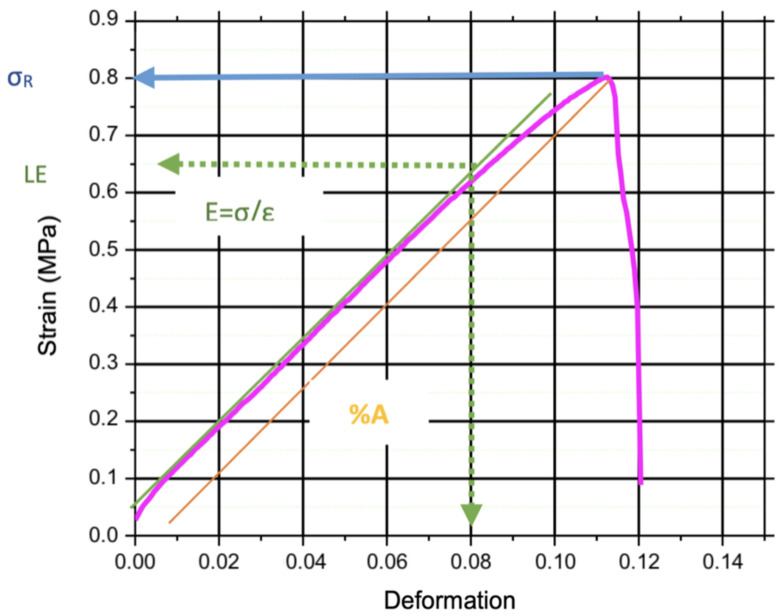
This figure shows the strain/deformation of banana peel film BM2. Including elastic modulus (*E*), ultimate tensile strength (*σ_R_*), elongation at break (*%A*), and the linear elastic (*LE*) region.

Figure 3 provides a more detailed analysis by illustrating the relationship between tension and deformation of BM2. A quasi-linear trend is observed in this graph, and the slope of this linear portion represents Young’s modulus or modulus of elasticity (*E*). This modulus quantifies the material’s stiffness and ability to deform under applied stress. The observed quasi-linear behavior indicates the elastic nature of the material, suggesting that it returns to its original form after the removal of the applied force. In this context, a calibration was performed on the linear section of the model, resulting in the determination of Young’s modulus of 7.48 MPa.

Furthermore, the graph reveals a notable feature—the material’s maximum tension, often denoted as the elastic limit (*LE*) or yield strength (*σy*), is reached at 0.65 MPa. Traditionally, beyond this point, one would expect a curved region indicating plastic deformation. However, in the case of BM2, this transition is almost imperceptible due to the material’s tendency to break abruptly once the elastic limit is reached.

It is important to point out that the mechanical tests that were carried out provided valuable data on the strength, flexibility, and overall behavior of the biopolymeric films. It turns out that the quality of these films depends significantly on their physical appearance, that is, their morphology. For example, when these films exhibit lumps or remnants of material that do not dissolve well, their resistance tends to decrease [39]. For these films to be strong and efficient, we need to improve their structure from the beginning of their production to be more uniform and flawless. Thus, they will not only be more resistant but will also be able to better adapt to different uses.

### 3.4. Morphology

This observation had significant implications for both the mechanical and moisture absorption properties, influencing the overall performance of the biopolymeric film. 

**Figure 4:**
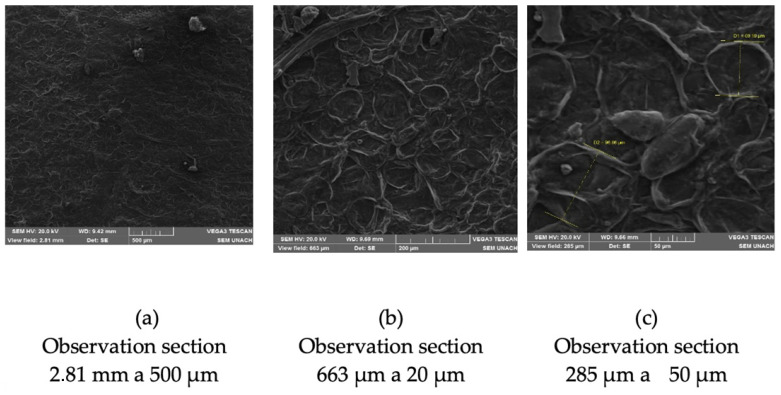
SEM micrographs of the biopolymeric banana peel film BM2. (**a**) General view with an irregular surface. (**b**) Fibrillar structure with cellulose accumulations. (**c**) Detail of fibers and discontinuities in the material matrix.

The irregular surface of the membrane (Figure 4) was attributed to the residual presence of insoluble fibrous content (crude fiber) or fat [39] present in banana peels. The fibrous waste introduced variations in the film’s texture, creating agglomerations that were visibly apparent in the micrographs.

The presence of these cellulose fibers directly impacted the biopolymeric membrane’s mechanical resistance. As elucidated by [30], irregularities, particularly in the form of agglomerations, contribute to a reduction in mechanical strength. The fibers acted as points of weakness, disrupting the homogeneity of the film structure and leading to localized stress concentrations. Consequently, this compromised mechanical strength may have affected the film’s performance in applications where robustness is critical.

The gelatinization process is key during the production of biopolymers; however, it is influenced by the amount of amylose and amylopectin present in starch. Amylose constitutes the amorphous part, while amylopectin forms the crystalline part of starch. Banana peel contains 82.84% amylose and 17.16% amylopectin [40], as well as 5.54% fat [15]. The presence of amylose leads to the formation of complexes with fat that are difficult to separate during gelatinization [20], resulting in an amorphous mixture with amylose residues, leading to films with irregular structures.

### 3.5. Thermogravimetric Analysis (TGA)

**Figure 5:**
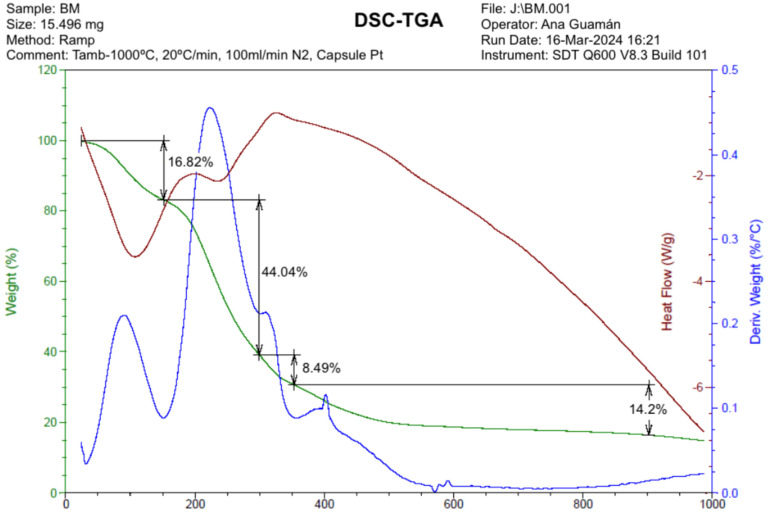
Thermogravimetric (TG) and derivative thermogravimetric (DTG) curves of banana peel film (BM2), showing its thermal degradation profile, weight loss stages, and decomposition temperatures under a controlled nitrogen atmosphere.

The thermogravimetric curve (TG) presented in Figure 5, along with its derivative (DTG), offers a comprehensive insight into the thermal degradation behavior of the biopolymeric film derived from banana peel waste (BM2). The detailed analysis of these curves reveals distinct stages of mass loss, shedding light on the film’s thermal stability and decomposition processes.

The thermal analysis of the BM2 biopolymeric membrane was conducted using 15.50 mg of the sample under an inert atmosphere (N_2_), following the ramp method, where the temperature was increased from 20 °C to 1000 °C at a heating rate of 30 °C/min.

The DSC-TGA technique (Differential Scanning Calorimetry–Thermogravimetric Analysis) allowed for the simultaneous measurement of mass changes (TGA) and heat flows (DSC) in the sample as a function of temperature.

The TGA curve (green line) shows a progressive decrease in mass as the temperature increases. The first mass loss was 16.02%, the second 44.04%, the third 8.49%, and the last 14.20%. Each stage likely corresponds to the degradation of a different polymer component or the release of volatiles. The total weight loss is the sum of the percentages from each stage.

The most significant weight loss, observed at 300 °C, corresponds to the mass of the starch-glycerin mixture. This stage involves the elimination of hydrogen bonds and the decomposition of polymer chains within the starch component of the film. Studies by [41,42] support this interpretation, highlighting the thermal degradation of starch-based materials in a similar temperature range.

The DSC curve corresponding to heat flow (red line) shows endothermic peaks (downward) and exothermic peaks (upward) during the thermal transitions of the biopolymer. Endothermic peaks correspond to fusion, dehydration, or glass transitions, while exothermic peaks may correspond to crystallization or exothermic decomposition reactions.

The graph shows an endothermic peak at approximately 110 °C, indicating that this is the melting point of the biopolymer. Additionally, two exothermic peaks are observed, the first at 200 °C and the second at 325 °C, suggesting that the structure does not exhibit completely crystalline characteristics.

The DTG curve (Derivative of Weight Loss, blue line) shows the weight loss rate as a function of temperature. The peaks in the DTG curve correspond to the points of the maximum decomposition rate.

### 3.6. Summary, Limitations, and Future Work 

#### 3.6.1. Low Tensile Strength and High Solubility

The low tensile strength observed in the biopolymeric membrane is an important characteristic that should be considered. However, this property can be strategically utilized in specific applications, such as food packaging, where flexibility is a key requirement [43,44]. Expressly, certain types of packaging incorporate essential oils, such as melaleuca and cinnamon, to reduce the film’s elastic modulus, enhancing its flexibility [10]. Additionally, using plasticizers in starch and cellulose-based films further improves their elongation and mechanical adaptability, making them more suitable for food industry applications.

The high solubility in water further emphasizes the film’s biodegradable nature, aligning with the global trend towards sustainable and environmentally friendly materials [45]. 

#### 3.6.2. High Water Absorption and Biodegradability 

The significant degree of water absorption underscores the film’s high biodegradability. This property positions the biopolymeric membrane as a viable alternative in applications where materials with minimal environmental impact are sought. The film’s ability to absorb water contributes to its eco-friendly profile, making it a promising solution for specific applications in disposable and environmentally sensitive contexts. The research conducted by [10] highlights the ecological profile and environmental benefits this type of membrane provides in the environmental context.

#### 3.6.3. Morphological Irregularities and Low Resistance 

To enhance biopolymeric films’ mechanical strength and functionality, it is fundamental to understand their surface structure and composition in detail. The use of techniques such as scanning electron microscopy (SEM) and energy-dispersive X-ray spectrometer (EDS) analysis allows for an in-depth examination of the film’s surface topography, identifying irregularities and insoluble materials like cellulose fibers, which can weaken the structure and affect the film’s mechanical strength and its ability to resist moisture absorption. This aspect is crucial in applications that require maintaining low moisture levels, such as packaging sensitive products. 

To overcome these challenges, optimizing the production process and adjusting the hydrolysis of cellulose improves cellulose solubility, resulting in a more uniform and defect-free film surface. These improvements increase the film’s strength and durability and optimize its moisture barrier properties, expanding its applicability in various situations, from packaging to structural applications where robustness and moisture resistance are key. The study conducted by [9] also recommends extending the shelf life and maintaining the quality of packaged products, especially moisture-sensitive foods, by optimizing biopolymeric films for use in packaging.

#### 3.6.4. The Thermogravimetric Analysis

The study allows observation of how the biopolymeric film undergoes weight loss as a function of increasing temperature. This information is critical for understanding the material’s thermal decomposition, degradation points, and the overall thermal stability of the film.

By subjecting the biopolymeric film to thermal analysis, deep insights into its behavior in extreme temperature environments can be obtained. These findings are particularly valuable for applications where exposure to temperature variations is anticipated, such as in packaging and transportation or in any other scenario where thermal stability is critical. In this context, [8] highlights the importance of the weight loss of the biopolymeric film as a function of increasing temperature; their thermal analysis is found in the section discussing the thermal degradation and thermal stability of biopolymers.

#### 3.6.5. Future Work and Applications

While the biopolymeric film exhibits limitations, such as low tensile strength and irregular morphology, these characteristics can be optimized for specific applications such as food packing. Future research and development efforts could focus on refining the film’s formulation, addressing morphological irregularities, and exploring novel applications where its unique combination of properties, including high biodegradability and UV-blocking capability, can be harnessed effectively, as demonstrated by [46] in their research.

The overall findings emphasize the sustainability implications of the biopolymeric membrane BM2. Its low environmental impact, high biodegradability, and potential applications in UV protection align with the growing emphasis on sustainable materials in various industries, a fact also supported by [46] in their research. Future endeavors in this domain can contribute to the shift towards eco-conscious practices in material science and product development.

## 4. Conclusions

The present project developed and characterized a biopolymeric membrane derived from mature banana peel waste (BM2) and evaluated its water absorption, solubility, mechanical properties, morphology, and thermal stability. The findings revealed that BM2 exhibits notable hydrophilic behavior, with high water absorption (115.23%) and solubility (61.75%), making it highly biodegradable and requiring structural improvements for practical applications. The mechanical analysis demonstrated a low tensile strength of 0.8 MPa, attributed to the presence of undissolved cellulose fibers, as evidenced by SEM analysis, which showed irregularities in the film’s surface. Thermogravimetric analysis (TGA) confirmed the thermal degradation range between 160 °C and 300 °C, indicating moderate thermal stability. Despite these mechanical challenges, the UV-visible spectrophotometry results showed promising optical properties, suggesting its potential for biodegradable food packaging and coatings. The findings position BM2 as a sustainable alternative to synthetic plastics, aligning with the growing global demand for eco-friendly materials.

Future works and research will be focused on enhancing BM2’s mechanical strength and water resistance through crosslinking techniques, nanocomposite reinforcement, or hydrophobic modifications to improve its structural integrity in high-moisture environments. Additionally, the optimization of plasticizer content, cellulose dissolution, and controlled drying techniques could lead to more uniform films with enhanced properties. Also, it is necessary to explore the incorporation of antimicrobial agents or bioactive compounds to expand their applications in active food packaging and biomedical fields. Scaling up production and conducting long-term biodegradation and storage stability tests will be crucial to validating its industrial feasibility. 

## Figures and Tables

**Table 1:** Comparison Table–Water Absorption and Solubility (%).

Sample	Water Absorption (%)	Solubility (%)	Reference
BM2	115.23	61.75	This study
banana pseudo stem-based film	4.6	40	Abera et al., 2024 [17]
*wak* banana peel starch film	231.5	--	Irmayanti & Anwar, 2024 [19]
banana peel and corn starch film	60.65	--	Sultan & Johari, 2017 [32]
Banana peel paste-corn starch film	92–113	31–36	Verma, et al., 2022 [28]

## Data Availability

The original contributions presented in the study are included in the article, and further inquiries can be directed to the corresponding authors.
